# Predictors of the Uptake of A (H1N1) Influenza Vaccine: Findings from a Population-Based Longitudinal Study in Tokyo

**DOI:** 10.1371/journal.pone.0018893

**Published:** 2011-04-27

**Authors:** Siyan Yi, Daisuke Nonaka, Marino Nomoto, Jun Kobayashi, Tetsuya Mizoue

**Affiliations:** 1 Department of Epidemiology and International Health, International Clinical Research Center, National Center for Global Health and Medicine, Tokyo, Japan; 2 Department of Community and Global Health, School of International Health, Graduate School of Medicine, The University of Tokyo, Tokyo, Japan; 3 Bureau of International Cooperation, National Center for Global Health and Medicine, Tokyo, Japan; University of Hong Kong, Hong Kong

## Abstract

**Background:**

Overall pandemic A (H1N1) influenza vaccination rates remain low across all nations, including Japan. To increase the rates, it is important to understand the motives and barriers for the acceptance of the vaccine. We conducted this study to determine potential predictors of the uptake of A (H1N1) influenza vaccine in a cohort of Japanese general population.

**Methodology/Principal Findings:**

By using self-administered questionnaires, this population-based longitudinal study was conducted from October 2009 to April 2010 among 428 adults aged 18–65 years randomly selected from each household residing in four wards and one city in Tokyo. Multiple logistic regression analyses were performed. Of total, 38.1% of participants received seasonal influenza vaccine during the preceding season, 57.0% had willingness to accept A (H1N1) influenza vaccine at baseline, and 12.1% had received A (H1N1) influenza vaccine by the time of follow-up. After adjustment for potential confounding variables, people who had been vaccinated were significantly more likely to be living with an underlying disease (*p* = 0.001), to perceive high susceptibility to influenza (*p* = 0.03), to have willingness to pay even if the vaccine costs ≥ US$44 (*p* = 0.04), to have received seasonal influenza vaccine during the preceding season (*p*<0.001), and to have willingness to accept A (H1N1) influenza vaccine at baseline (*p*<0.001) compared to those who had not been vaccinated.

**Conclusions/Significance:**

While studies have reported high rates of willingness to receive A (H1N1) influenza vaccine, these rates may not transpire in the actual practices. The uptake of the vaccine may be determined by several potential factors such as perceived susceptibility to influenza and sensitivity to vaccination cost in general population.

## Introduction

A new swine-origin influenza A (H1N1) emerged in early 2009 in Mexico and the United States and has since spread worldwide [1]. On June 11, 2009, the World Health Organization (WHO) declared the disease to be a pandemic phase 6, and the world moved into the first global influenza pandemic in more than 40 years [2]. As of March 01, 2010, laboratory-confirmed cases had been reported in more than 213 countries and territories with at least 18,449 deaths worldwide [3]. In Japan, the first outbreak of the novel influenza was confirmed in May 2009, and it became pandemic in November of the same year [4], [5]. This situation triggered an extensive public health response, which included large scale efforts to educate the general public about the pandemic, including benefits and risks of vaccination and to develop strategies to prevent transmission [6].

Vaccination is a primary public health measure to curb the spread of A (H1N1) influenza pandemic due to the lack of innate immunity as a result of the strain's novelty [7]. In Japan, the vaccination campaigns started in November 2009 targeting prioritized populations including people living with underlying diseases, pregnant women, children aged five years or younger, and elderly people aged 65 years or older [8]. The national standardized cost for the vaccination is approximately US$ 42 for the first dose and US$ 30 for the second dose. People in prioritized groups are, however, partially or fully subsidized by the local government. Vaccination is provided at most healthcare facilities such as hospitals, clinics, and public health centers. Local government is responsible for dissemination of information regarding the vaccination services through mass media such as newspapers, town papers, posters, and internet.

Early vaccination against the virus is cost-effective and may avert the deaths [9]. Public acceptance of the vaccination is thus a crucial factor in controlling the pandemic [10]. However, increasing the public acceptance of the vaccination may be more difficult than addressing the technical and scientific challenges involved in quickly producing large quantities of a safe and effective vaccine [11]. Considering that, despite unprecedented public education campaigns and a worldwide pandemic, only about 20% of adults in the United States have been vaccinated against the pandemic influenza [6]. When fear of A (H1N1) influenza was widespread, less than half of all adults were willing to get vaccinated [12], and almost 20% of adults said they would not consider getting vaccinated, even if people in their community were sick or dying from the pandemic influenza [13]. To increase A (H1N1) influenza vaccination rates, it is important to understand the motives and barriers for the acceptance of the vaccine.

Evidence has emerged regarding factors associated with willingness to get A (H1N1) influenza vaccination. However, only a few studies have examined the association of these factors with the actual uptake of the vaccine, and most of these studies have been conducted in key populations such as healthcare workers, school teachers, people living with underlying diseases, elderly people, and pregnant women. The identified factors consistently reported in these studies include personal experience of getting vaccinated against seasonal influenza [12], [14], [15], personal perception of high risk of getting infected by A (H1N1) influenza [15], [16], [17], attitudes towards vaccine efficacy and safety [6], [18], [19], [20], perceived barriers to get vaccinated [21], [22], and social norms regarding A (H1N1) influenza vaccination [21].

These findings are important in response to the need for revising or constructing a preparedness plan in early stage of the fight against the pandemic A (H1N1) influenza. However, findings from these studies are limited by the cross-sectional nature of the data. To the best of our knowledge, one longitudinal study has been conducted to explore the influencing factors related to the uptake of A (H1N1) influenza vaccine among school teachers in the United States [21]. To address this shortcoming, we conducted this study to determine potential predictors of the actual uptake of A (H1N1) influenza vaccination in a cohort of Japanese general population.

## Methods

### Ethics statement

We sent a postal mail in which an information sheet and a questionnaire were included to each selected person. In the information sheet, we fully explained the nature and possible consequences of the study and requested them to note if they were willing to participate in the study. For those who did not want to participate, we requested them to indicate on the sheet and return it to us. The study protocol was reviewed and approved by the Ethics Committee of the National Center for Global Health and Medicine, Tokyo, Japan.

### Sampling procedure

Baseline study was conducted in October 2009 and data for follow-up was collected in April 2010. We used multi-stage sampling strategy to select participants for this study. We first randomly selected four wards out of 23 wards and two cities out of 26 cities comprising metropolitan Tokyo. Of these two selected cities, one city refused participation. Number of towns in each ward and city ranges from 20 to 155, and approximately 10% of the towns were randomly selected. Probability proportional to size sampling was used to select adults aged 18–65 years randomly from the residential registries obtained from the municipal offices.

**Figure 1:**
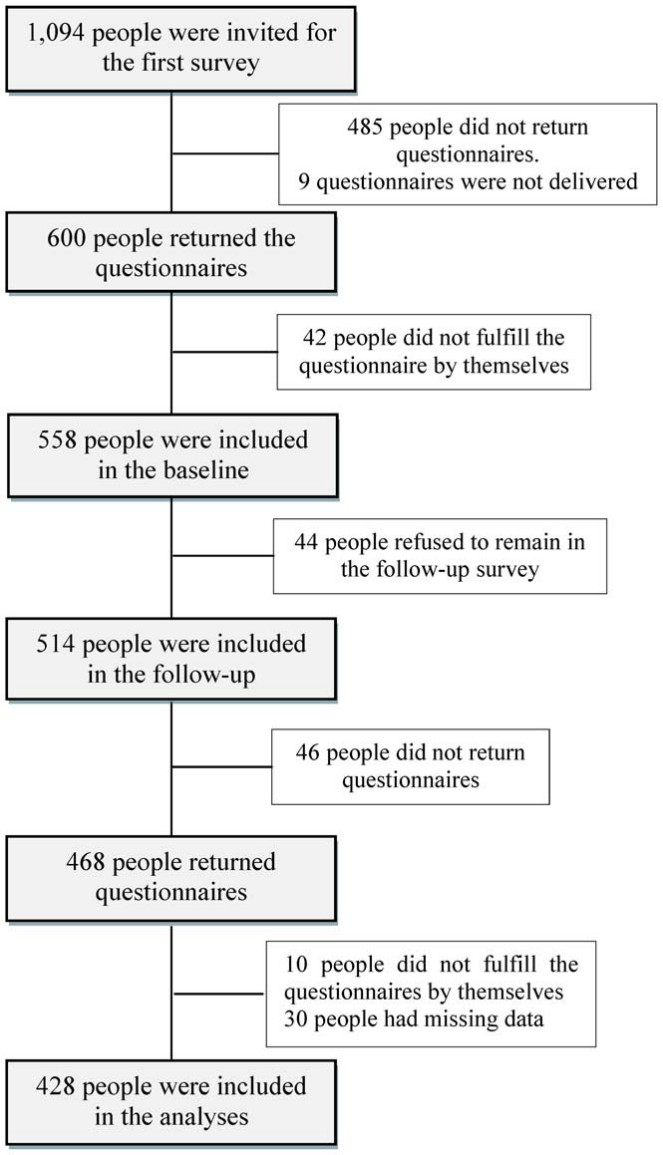
Flow of enrollment and retention of the study participants.

Figure 1 shows the flow of enrollment and retention of the study participants. Out of 1,094 questionnaires distributed at baseline survey, 600 were completed and returned, giving a response rate of 54.8%. We further excluded 42 people who did not complete the questionnaires by themselves, and 558 (51.0%) respondents were included in baseline survey. Of these, 44 people declined to remain in the cohort for follow-up. We invited 514 people for follow-up, and 468 (42.8%) completed and returned the questionnaires. We further excluded 30 people with missing data in any measures and 10 people who did not complete the questionnaires by themselves. We finally included 428 (39.1%) people in the analyses.

### Questionnaires and measurements

Self-administered, anonymous questionnaires were mailed to participants in both baseline and follow-up survey by using the same study protocol. The survey questionnaires were developed based on measures adapted from previous studies and guidelines recommended by the Japanese government and non-government agencies. In baseline survey, we collected information regarding socio-demographic characteristics, history of living with underlying diseases, history of living with people in high risk groups, history of receiving seasonal influenza vaccine in 2008–2009, history of seasonal influenza infection during the current season (2008–2009), knowledge on A (H1N1) influenza and its vaccine, perception of risk of getting infected with A (H1N1) influenza, perception of seriousness of pandemic A (H1N1) influenza, preventive behavior against A (H1N1) influenza, attitudes towards A (H1N1) influenza and its vaccine, and willingness to accept vaccination against A (H1N1) influenza. In follow-up survey, we additionally collected information regarding history of influenza infection during the current season (2009–2010) and history of the uptake of vaccine against seasonal influenza and A (H1N1) influenza during the current season (2009–2010).

Willingness to accept vaccination against A (H1N1) influenza was assessed by asking: “Do you plan to get A (H1N1) influenza vaccine during this fall or winter if it becomes available?” with three response options including “yes,” “no,” and “not sure.” The uptake of influenza vaccine was assessed by asking the following yes/no questions: “Did you receive seasonal influenza vaccine during the last season (2008–2009)?”; “Did you receive seasonal influenza vaccine during this season (2009–2010)?”; and “Did you receive new swine A (H1N1) influenza vaccine during this season (2009–2010)?” To assess risk perception, we asked: “Do you think you are constitutionally susceptible to influenza infection?” using a 4-point scale that included “very high,” “high,” “not so high,” and “not high at all.” Regarding attitude towards vaccination, participants were asked whether they feel anxious about the side effects of swine A (H1N1) influenza vaccine with “a lot,” “some,” “not much,” and “not at all” as response choices. To assess willingness to pay for the vaccine, we asked: “Up to how much would you pay for receiving swine A (H1N1) influenza vaccine?” with four response options including “free of charge,” “US$ 1 to <22,” “US$ 22 to <44,” and “≥ US$ 44.”

Regarding knowledge on A (H1N1) influenza and its vaccine, we evaluated the correct responses to four yes/no questions regarding modes of transmission, the fact that there have been healthy people who had died from A (H1N1) influenza, people at risk of A (H1N1) influenza, effectiveness of antiviral medicines such as Tamiflu and Relenza against A (H1N1) influenza, and side effects of vaccine against A (H1N1) influenza. To assess participants' preventive behavior, we asked whether participants washed their hands after returning home during the preceding week (yes/no).

For socio-demographic characteristics, participants reported their age (continuous), gender (male or female), education attainment (secondary/high school, college, or university/graduate school), marital status (unmarried or married), employment status (employed or unemployed), annual household income (<US$ 22,000, US$ 22,000– <55,000, US$ 55,000–111,000, ≥ US$ 111,000), and whether the participant was living with children who go to school (yes/no) and with people in high risk groups such as people with an underlying disease, pregnant women, children aged five years or younger, and elderly people aged 65 years or older (yes/no). In this study, underlying diseases included chronic respiratory diseases, chronic metabolic diseases, chronic heart diseases, kidney diseases, and immunodeficiency diseases.

### Data analyses

We compared characteristics of participants at baseline and follow-up and characteristics of people who successfully completed the study to those of people who participated in baseline but refused to remain in the cohort or did not return the questionnaires at follow-up by using χ^2^ test or Fisher's Exact test for categorical variables and student *t*-test for continuous variables. Using cross tabulations, we assessed association between the uptake of A (H1N1) influenza vaccine with several characteristics of participants. We tested statistical significance of the association by using Pearson's χ^2^ test or Fisher's exact test.

A multiple logistic regression model was then constructed to determine potential predictors of the uptake of A (H1N1) influenza vaccine. Socio-demographic characteristics and variables on vaccination history, knowledge, perception, and behavior were included in the model if they were associated with the uptake of A (H1N1) influenza vaccine at a level of *p*≤0.10 in bivariate analyses. Two-sided *p*-values of <0.05 were regarded as statistically significant. We used SPSS version 17.0 (SPSS Inc, Chicago, IL) for all the statistical analyses.

## Results

### Characteristics of participants

Characteristics of the participants at baseline and follow-up are shown in Table 1. Of total, 58.9% were female with mean age of 42.6 years (SD  = 11.9 years). More than half (55.4%) of participants had annual household income of ≥ US$55,000, 60.7% were married, 48.4% lived with people in high-risk groups, and 9.6% had at least one underlying disease. Regarding vaccination history, 38.1% received seasonal influenza vaccine during the preceding season (2008–2009), 57.0% had willingness to accept A (H1N1) influenza vaccine at baseline, and 12.1% had been vaccinated by the time of follow-up. As shown in Table 1, no significant difference was found in comparisons between characteristics of respondents at baseline and follow-up. People who completed the study (*n* = 428) were significantly more likely to have attained higher education (*p* = 0.03) and to live with people in high-risk groups (*p* = 0.009) compared to those who were lost to follow-up (n = 90).

**Table 1:** Characteristics of the participants at baseline and follow-up.

	Baseline (*n* = 558)		Follow-up (*n* = 428)	
Characteristics	*n*	*%*	*n*	*%*
Gender				
Male	328	58.8	252	58.9
Female	230	41.2	176	41.1
Mean age in years (SD)	42.9 (12.5)		42.6 (11.9)	
Marital status				
Unmarried	222	40.0	168	39.3
Married	333	60.0	260	60.7
Employment status				
Employed	436	78.4	334	78.0
Unemployed	120	21.6	94	22.0
Education attainment				
Secondary/high school	189	34.4	161	37.6
College	136	24.7	106	24.8
University or higher	225	40.9	161	37.6
Annual household income (US$)*				
<22,000	65	11.9	46	10.7
22,000– <55,000	192	35.2	145	33.9
55,000– <111,000	214	39.2	173	40.4
≥111,000	74	13.7	64	15.0
Having underlying diseases†				
Yes	57	10.3	41	9.6
No	499	89.7	387	90.4
Living with high-risk groups‡				
Yes	258	46.3	207	48.4
No	298	53.5	221	51.6
Living with school-going children§				
Yes	193	34.6	153	35.7
No	363	65.2	275	64.3
History of influenza infection in 2009–2010				
Yes	-	-	16	3.7
No	-	-	412	96.3

SD denotes standard deviation.

*Exchange rate: US$ 1  =  ¥90.

† Underlying diseases included chronic respiratory diseases, chronic metabolic diseases, chronic heart diseases, liver diseases, kidney diseases, and immunodeficiency diseases.

‡ High-risk groups included people with an underlying disease, pregnant women, children age five years or younger, and elderly people aged 65 years or older.

§ School-going children included primary school students to university students.

### Bivariate analysis results

Table 2 and Table 3 show bivariate association of the uptake of A (H1N1) influenza vaccine by the time of the follow-up with socio-demographic characteristics and knowledge, attitudes, and behavior toward A (H1N1) influenza and its vaccine. The prevalence of the vaccination uptake was significantly higher among women, people living with an underlying disease, people with higher family income, people who perceived high susceptibility to influenza, people who knew about the possible side effects of A (H1N1) influenza vaccine, people who were willing to pay even if the vaccine costs ≥ US$ 44, people who received seasonal influenza vaccination during the preceding season (2008–2009), and people who had willingness to accept A (H1N1) influenza vaccine at baseline, relative to their comparison groups.

**Table 2:** Bivariate association between participant's characteristics and the uptake of A (H1N1) influenza vaccine.

	Total (*n* = 428)	Vaccine receivers (*n* = 52)	
Characteristics	*n*	*n* (%)	*p*-value*
**Socio-economic status**			
Gender			
Male	176	14 (8.0)	0.03
Female	252	38 (15.1)	
Age (years)			
18–39	169	22 (13.0)	0.91
40–59	207	24 (11.6)	
60–64	52	6 (11.5)	
Marital status			
Unmarried	168	18 (10.7)	0.47
Married	260	34 (13.1)	
Education attainment			
Secondary/high school	161	16 (9.9)	0.49
College	106	13 (12.1)	
University or higher	161	23 (14.3)	
Employment status			
Employed	334	15 (16.0)	0.20
Unemployed	94	37 (11.1)	
Annual household income (US$)			
<22,000	46	5 (10.9)	0.005
22,000– <55,000	145	17 (11.7)	
55,000– <111,000	173	14 (8.1)	
≥111,000	64	16 (25.0)	
Having underlying diseases†			
Yes	41	12 (29.3)	<0.001
No	387	40 (10.3)	
Living with high-risk groups‡			
Yes	207	31 (15.0)	0.08
No	221	21 (9.5)	
Living with school-going children§			
Yes	153	16 (10.5)	0.42
No	275	36 (13.1)	

*p-values were based on χ^2^ test or Fisher's Exact test.

† Underlying diseases included chronic respiratory diseases, chronic metabolic diseases, chronic heart diseases, liver diseases, kidney diseases, and immunodeficiency diseases.

‡ High-risk groups included people with an underlying disease, pregnant women, children age five years or younger, and elderly people aged 65 years or older.

§ School-going children included primary school students to university students.

**Table 3:** Knowledge, attitudes, and behavior towards A (H1N1) influenza and its vaccine among vaccine receivers and non-receivers.

	Total (*n* = 428)	Vaccine receivers (*n* = 52)	
Characteristics	*n*	*n* (%)	*p*-value*
**Knowledge on A (H1N1) influenza**			
Modes of transmission			
Correct	375	51 (13.6)	0.56
Incorrect	53	8 (15.1)	
The fact that there have been healthy people who have died from A (H1N1) influenza			
Correct	333	42 (12.6)	0.58
Incorrect	95	10 (10.5)	
People at risk of A (H1N1) influenza			
Correct	416	51 (12.3)	0.56
Incorrect	12	1 (8.3)	
Effectiveness of antiviral medicine such as Tamiflu or Relenza			
Correct	324	41 (12.7)	0.59
Incorrect	103	11 (10.7)	
**Perception**			
Perceived susceptibility to influenza			
Very high/high	95	21 (22.1)	0.001
Not so high/not high at all	333	31 (9.3)	
**Preventive behavior**			
Washing hand after returning home in the past week			
Yes	377	43 (11.4)	0.20
No	51	9 (17.6)	
**Knowledge, attitudes, and behavior towards influenza vaccine**			
Knowledge about possible side effects of A (H1N1) influenza vaccine			
Yes	303	44 (14.5)	0.02
No	125	8 (6.4)	
Anxiety about adverse effects of A (H1N1) influenza vaccine			
A lot/some	274	36 (13.1)	0.40
Not much/not at all	154	16 (10.4)	
Willingness to pay for vaccine if it costs (US$)			
Free of charge	44	2 (4.5)	0.006
1– <22	160	15 (9.4)	
22– <44	179	23 (12.8)	
≥44	45	12 (26.7)	
Receiving seasonal influenza vaccination during the last season (2008–2009)			
Yes	163	41 (25.2)	<0.001
No	265	11 (4.2)	
Willing to accept A (H1N1) influenza vaccine if it is available			
Yes	244	43 (17.6)	<0.001
No	184	9 (4.9)	

*p-values were based on χ^2^ test or Fisher's Exact test.

### Multivariate analysis results

As shown in Table 4, after adjustment for potential confounding variables, people who had been vaccinated were significantly more likely to live with an underlying disease (*p* = 0.001), to perceive high personal susceptibility to influenza (*p* = 0.03), to have willingness to pay for the vaccine even if it costs ≥ US$ 44 (*p* = 0.04), to have received seasonal influenza vaccination during the preceding season (2008–2009) (*p*<0.001), and to have willingness to accept A (H1N1) influenza vaccine at baseline (*p*<0.001) compared to those who had not been vaccinated.

**Table 4:** Factors associated with the uptake of A (H1N1) influenza vaccine in multivariate logistic regression model.

Characteristics	Adjusted OR* (95% CI)	*p*-value
**Socio-economic status**		
Gender		
Male	2.04 (0.97–4.28)	0.06
Female	Reference	
Annual household income (US$) †		
<22,000	Reference	
22,000– <55,000	1.21 (0.40–3.71)	0.73
55,000– <111,000	0.73 (0.23–2.35)	0.60
≥111,000	2.82 (0.81–9.93)	0.10
Having underlying diseases ‡		
Yes	4.43 (1.90–10.33)	0.001
No	Reference	
Living with high-risk groups §		
Yes	1.34 (0.71–2.54)	0.37
No	Reference	
**Perception**		
Perceived susceptibility to influenza		
Very high/high	2.67 (1.12–6.37)	0.03
Not so high/not high at all	Reference	
**Knowledge, attitudes, and behavior towards influenza vaccine**		
Knowledge about possible side effects of A (H1N1) influenza vaccine		
Yes	Reference	
No	0.51 (0.22–1.16)	0.11
Willing to pay for vaccine if it costs (US$)		
Free of charge	Reference	
1– <22	1.80 (0.36–8.87)	0.47
22– <44	2.33 (0.47–11.51)	0.30
≥44	5.99 (1.07–33.46)	0.04
Receiving seasonal influenza vaccine during the last season (2008–2009)		
Yes	7.33 (3.46–15.55)	<0.001
No	Reference	
Willing to accept A (H1N1) influenza vaccine if it is available		
Yes	4.27 (1.94–9.39)	<0.001
No	Reference	

OR denotes odds ratio; CI denotes confidence interval.

* Other variables in the model included age, education attainment, marital status, employment status, and anxiety about adverse effects of A (H1N1) influenza vaccine.

† Exchange rate: US$ 1  =  ¥90.

‡ Underlying diseases included chronic respiratory diseases, chronic metabolic diseases, chronic heart diseases, liver diseases, kidney diseases, and immunodeficiency diseases.

§ High-risk groups included people with an underlying disease, pregnant women, children age five years or younger, and elderly people aged 65 years or older.

## Discussion

This study represents a few attempts to identify influencing factors associated with the actual uptake of A (H1N1) influenza vaccine using a longitudinal design in general population. Several potential predictors have been explored including willingness to accept A (H1N1) influenza vaccine at baseline, receiving seasonal influenza vaccine during the preceding season (2008–2009), willingness to pay for the vaccine even if it costs ≥ US$ 44, perceived high susceptibility to influenza, and living with an underlying disease.

People who had willingness to accept A (H1N1) influenza vaccine at baseline were more than four times more likely to get vaccinated compared to those who had no willingness. However, among people who were willing to get a shot of the vaccine at baseline, only 17.6% had actually received it by the time of the follow-up. This finding may raise concerns in respect to other potential factors that might have diminished people's final decision making on whether to take the vaccination at different stages of the pandemic. Because baseline survey of this study was conducted when A (H1N1) influenza was at its peak in Japan, we can expect that people's willingness to get vaccinated was strongly anchored and well-defined in general population. The willingness might, however, be over-estimated, and people may change their mind when the pandemic situation become less severe and clinical evidence regarding efficacy and safety of the vaccine remained unavailable when vaccination campaigns were launched. In Japan, A (H1N1) influenza vaccine was not available for general population until January 2010 when the number of A (H1N1) influenza cases was going down [23].

In line with previous studies [12], [24], our results show that the receipt of the novel A (H1N1) influenza vaccine were strongly predicted by seasonal influenza vaccination in the preceding season, suggesting common attitudinal motives and barriers to both vaccines. The public is likely to share common concerns regarding the pandemic and seasonal influenza vaccination, particularly in the areas of vaccine safety and side effects and personal risks [25]. Strategies to improve the uptake of seasonal influenza vaccine by general population should therefore be adapted as a part of the pandemic preparedness plan, as dissemination of correct information regarding vaccination may be more difficult at the time of crisis [26].

Among reasons for the low A (H1N1) influenza vaccine coverage rates, there may have been a lack of concern about the individual risk, which may translate into a lack of willingness or urgency to get vaccinated, particularly if there is mistrust of information provided by public health or government authorities [25]. There may have also been confusion regarding the differences between the pandemic and seasonal influenza as well as their vaccines [24]. People may believe that A (H1N1) influenza is as mild as seasonal influenza, and its vaccine may be necessary only for people in high risk groups. Without clarification of the misunderstanding, people might see A (H1N1) influenza as a relatively mild disease and think that it may not worth the risk to get vaccinated as the vaccine has not been thoroughly tested for efficacy and safety.

People may perform a sort of trade-off between perceived risk and perceived benefit regarding the vaccine uptake. In this study, the majority (77.8%) of participants perceived their susceptibility to A (H1N1) influenza as “not so high” or “not high at all,” and those in this category were almost three times less likely to get vaccinated compared to those who perceived their susceptibility as “high or very high.” Meanwhile, more than two thirds of them were anxious about adverse effects of the vaccine. This suggests that perceived risks may exceed perceived benefit. In addition, the acceptability of A (H1N1) influenza vaccine was sensitive to cost, although Japan is a fairly affluent country with a high degree of vigilance for influenza. People who were willing to pay for the vaccine even if it costs ≥ US$ 44 were almost seven times more likely to get vaccinated compared to those who were willing to get vaccinated only if it is available for free. Such a cost sensitivity may also be true for the case of seasonal influenza vaccine for which, in Japan, people have to pay up to approximately US$ 25 to US$ 60 [8]. We may expect that this sensitivity could even be higher in developing countries and in those countries where people may feel less anxious about A (H1N1) influenza.

It is worth noting that some important factors, such as anxiety about adverse effects of A (H1N1) influenza vaccine and living with people in high risk groups, did not retain their significant association with the uptake of A (H1N1) influenza vaccine in this study. Concerns about vaccine safety and side effects have been consistently reported as a potential determinant of willingness to get vaccinated and/or the uptake of the vaccine [25], [27], while people living with high risk groups have been prioritized as a target group for vaccination campaigns [28]. These unexpected findings may be explained by the timing when the study was conducted, which was in a relatively later stage of A (H1N1) influenza vaccine progress, and the different definition of terms used in the current study. Our definition of “high-risk groups” covered a wide range of people including people living with underlying diseases, pregnant women, small children, and elderly people.

The major strengths of this study include the longitudinal design with the ability to document not only people's willingness to accept A (H1N1) vaccine but also their actual practice of receiving the vaccine in six-month follow-up. Moreover, we were able to control for the effects of several potential confounding factors.

Several limitations of this study should also be noticed. First, our findings were limited by the validity of self-reporting measures, which may lead to either under-reporting or over-reporting due to social desirability or conformity. Second, the relatively low response rate (54.8%) may have resulted in a biased sample and become potential threats to the generalizability of the findings to the whole population. This response rate, however, is comparable to or even higher than that of many mailing or telephone surveys in other countries [12], [16], [29], [30]. Third, although the measure of each construct was carefully developed, the unknown validity and reliability of the study instruments may be of concern, and could result in the difficulties in making cross-population comparisons. Forth, our relatively small sample size may not sufficiently powerful to detect modest association. The final limitation concerns the timing of the survey that might have led to both an overestimate of willingness to receive the vaccination and an underestimate of the vaccine coverage rate among Japanese adult population since the controversy about the efficacy, safety, and necessity of the pandemic A (H1N1) influenza vaccine was growing over the study period.

Despite these limitations, this study contributes to the literature in several ways. While studies have reported high rates of willingness to receive A (H1N1) influenza vaccine, these rates may not transpire in the actual practices among general population. The uptake of the pandemic A (H1N1) influenza vaccine may be determined by several potential factors such as perceived susceptibility to influenza and sensitivity to vaccination cost. Although cultural differences could affect the acceptance of vaccines in general [31], we believe that there are common motives and barriers to the uptake of A (H1N1) influenza vaccine that exist across geographical regions and racial groups. Findings of this study can therefore serve as a reference for the development of strategies to improve the uptake of the pandemic A (H1N1) influenza vaccine in general population in Japan as well as in other countries.
